# Effects of GLP-1 receptor agonists on body weight and cardiovascular outcomes: a review

**DOI:** 10.1007/s12471-026-02073-3

**Published:** 2026-09-14

**Authors:** Susan J. F. van Maren, Eveline P. M. van Doorn, Mark M. Smits, Cees J. J. Tack, Elisabeth F. C. van Rossum, Saloua El Messaoudi

**Affiliations:** 1 Department of Cardiology, Radboud University Medical Centre, Geert Grooteplein Zuid 10, 6525 Nijmegen, GA The Netherlands; 2 Department of Internal Medicine, Radboud University Medical Centre, Nijmegen, The Netherlands; 3 Department of Internal Medicine, Erasmus MC, University Medical Centre Rotterdam, Rotterdam, The Netherlands; 4 Obesity Center CGG, Erasmus MC, University Medical Centre Rotterdam, Rotterdam, The Netherlands

**Keywords:** GLP-1RA, Body Weight, Cardiovascular outcomes

## Abstract

Obesity is a rapidly increasing global health challenge that is strongly linked to the cardiorenometabolic syndrome, cardiovascular morbidity, and mortality. Lifestyle interventions remain the cornerstone of treatment, but average long-term weight loss is modest and durable weight maintenance is often limited. Bariatric surgery is not suitable for all patients, and substantial weight regain occurs in a considerable proportion over time. Glucagon-like peptide‑1 receptor agonists (GLP-1RAs), originally developed for type 2 diabetes (T2D), have emerged as potent agents for weight reduction and cardiometabolic risk improvement. By enhancing glucose-dependent insulin secretion, suppressing appetite, improving lipid metabolism, and exerting anti-inflammatory, endothelial, and haemodynamic effects, GLP-1RAs influence multiple pathways relevant to cardiovascular disease.

Across large cardiovascular-outcome trials, GLP-1RAs consistently reduce major adverse cardiovascular events (MACE), all-cause mortality, and heart-failure hospitalizations in individuals with T2D. In populations with overweight or obesity without diabetes, semaglutide, as shown in the SELECT trial, has demonstrated significant cardiovascular risk reduction, partly independent of weight loss. Overall, GLP-1RAs represent a therapeutic option for cardiovascular prevention across a broad spectrum of patients at high-risk for or with established CVD. This review provides a comprehensive overview of the pleiotropic cardiovascular effects associated with GLP-1RAs and discusses their potential integration into cardiovascular care.

## Introduction

Obesity represents a major global health challenge and is considered the fifth leading risk factor for mortality worldwide. According to the World Health Organisation, one in eight individuals worldwide was living with obesity in 2022, and its prevalence continues to rise [1].

Obesity is a chronic disease that is strongly associated with a wide range of cardiorenometabolic diseases, including dyslipidaemia, hypertension, type 2 diabetes (T2D), and atherosclerotic cardiovascular disease (ASCVD), as well as numerous other chronic diseases. A BMI above 25 kg/m^2^ is associated with a progressive increase in cardiovascular mortality risk for each additional 5 kg/m^2^, particularly for coronary heart disease and ischemic stroke [2].

Current treatment strategies for obesity focus on achieving weight loss through lifestyle interventions, pharmacotherapy, and bariatric surgery. While intentional weight loss has clear cardiovascular benefits, [2] substantial weight loss through lifestyle interventions is often limited, and long-term weight maintenance by lifestyle interventions remains one of the greatest challenges in obesity management due to metabolic, hormonal, and neural adaptations. Many individuals experience substantial weight regain after initial weight loss, and while bariatric surgery can effectively reduce body weight and cardiovascular risk, it is not suitable for all patients and is associated with procedural risks and potential long-term complications. These limitations underscore the need for safe and effective therapies that support durable weight reduction.

**Fig. 1:**
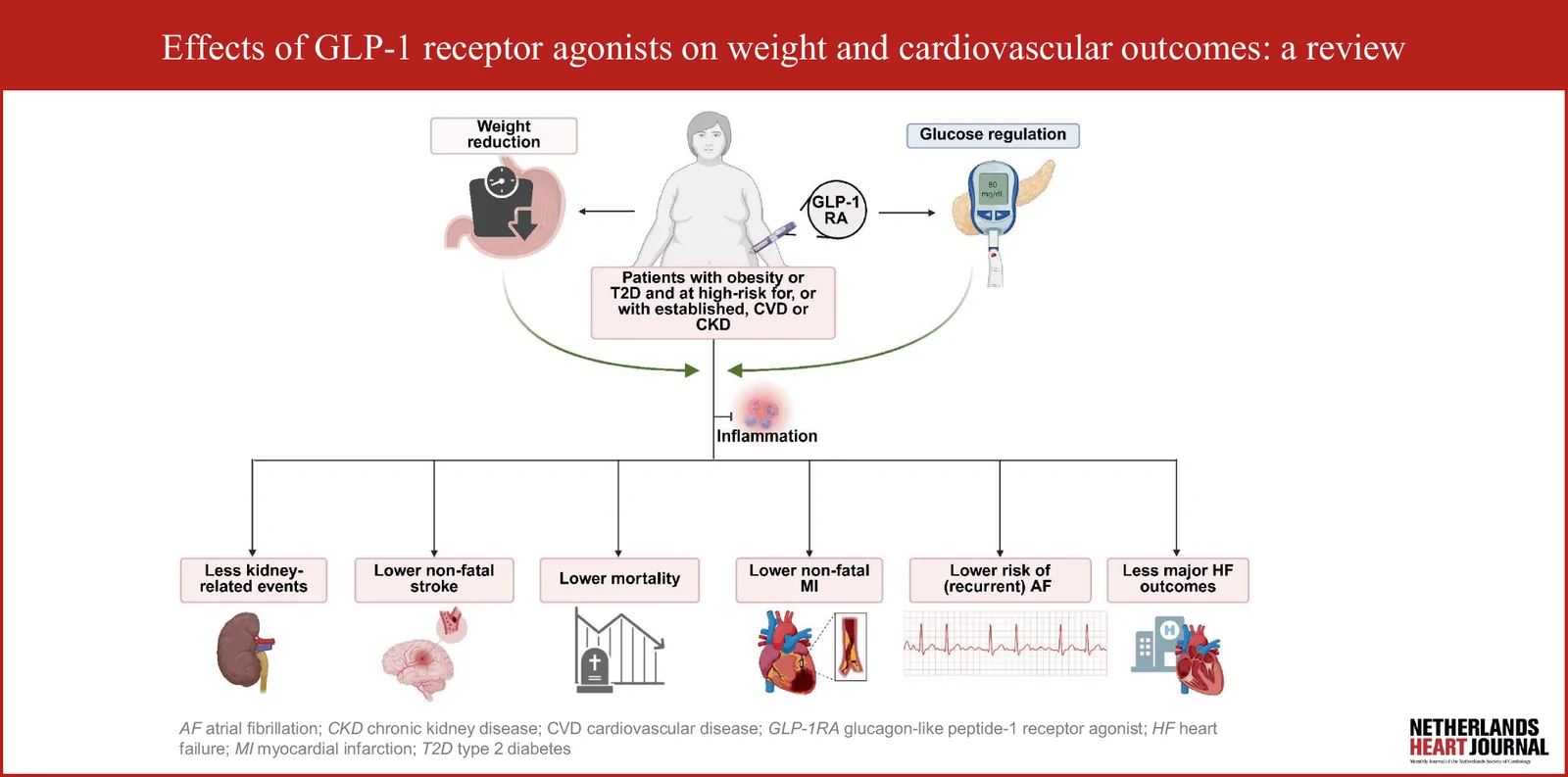
Infographic of the effects of GLP-1RAs on weight and cardiovascular outcomes

Glucagon-like peptide‑1 (GLP-1) receptor agonists (RAs) were initially developed for the treatment of T2D but have since gained widespread use for weight management and improvement of cardiorenometabolic health. Their favourable effects on body weight, glycaemic control, and cardiovascular risk factors have generated considerable interest in their potential to improve cardiovascular outcomes directly (Fig. 1).

The aim of this review is to provide an overview of the role of GLP-1RAs in weight management and cardiovascular outcomes among populations at high risk for or with established CVD.

## Physiology of GLP-1, GLP-1RAs and mechanism of action

Endogenous GLP‑1 is an incretin hormone secreted by intestinal L‑cells in response to nutrient intake, and is also produced by neurons within the brainstem. The receptor for GLP‑1 is widely distributed and is expressed not only in the pancreas, brain, and gastrointestinal tract but also in the lungs, kidney, cardiomyocytes, and vascular endothelial cells [3]. GLP‑1 regulates post-prandial glucose metabolism, satiety signalling, delayed gastric emptying, blood pressure reduction, and natriuresis [4]. However, endogenous GLP‑1 has a short half-life of approximately 1–2 min, as it is rapidly degraded by the enzyme dipeptidyl peptidase‑4 (DPP-4) and eliminated by the kidneys [5]. Less than 20% of secreted GLP‑1 reaches the systemic circulation. Consequently, the effects of endogenous GLP‑1 may not be due to direct activation of systemic GLP‑1 receptors but rather are mediated by vagal signaling [6]. This is in contrast to pharmacological GLP‑1 receptor agonists (GLP-1RAs), which directly activate GLP‑1 receptors in multiple tissues.

GLP-1RAs represent a structurally diverse class with distinct pharmacokinetics. The short-acting agents exenatide and lixisenatide are based on the exendin‑4 sequence, a naturally occurring peptide that escapes degradation by DPP‑4. Longer-acting agents include once-weekly exenatide, acylated human-sequence GLP-1RAs such as liraglutide and semaglutide, and fusion proteins such as dulaglutide. These structural distinctions influence absorption, receptor engagement, and tissue distribution [7].

**Fig. 2:**
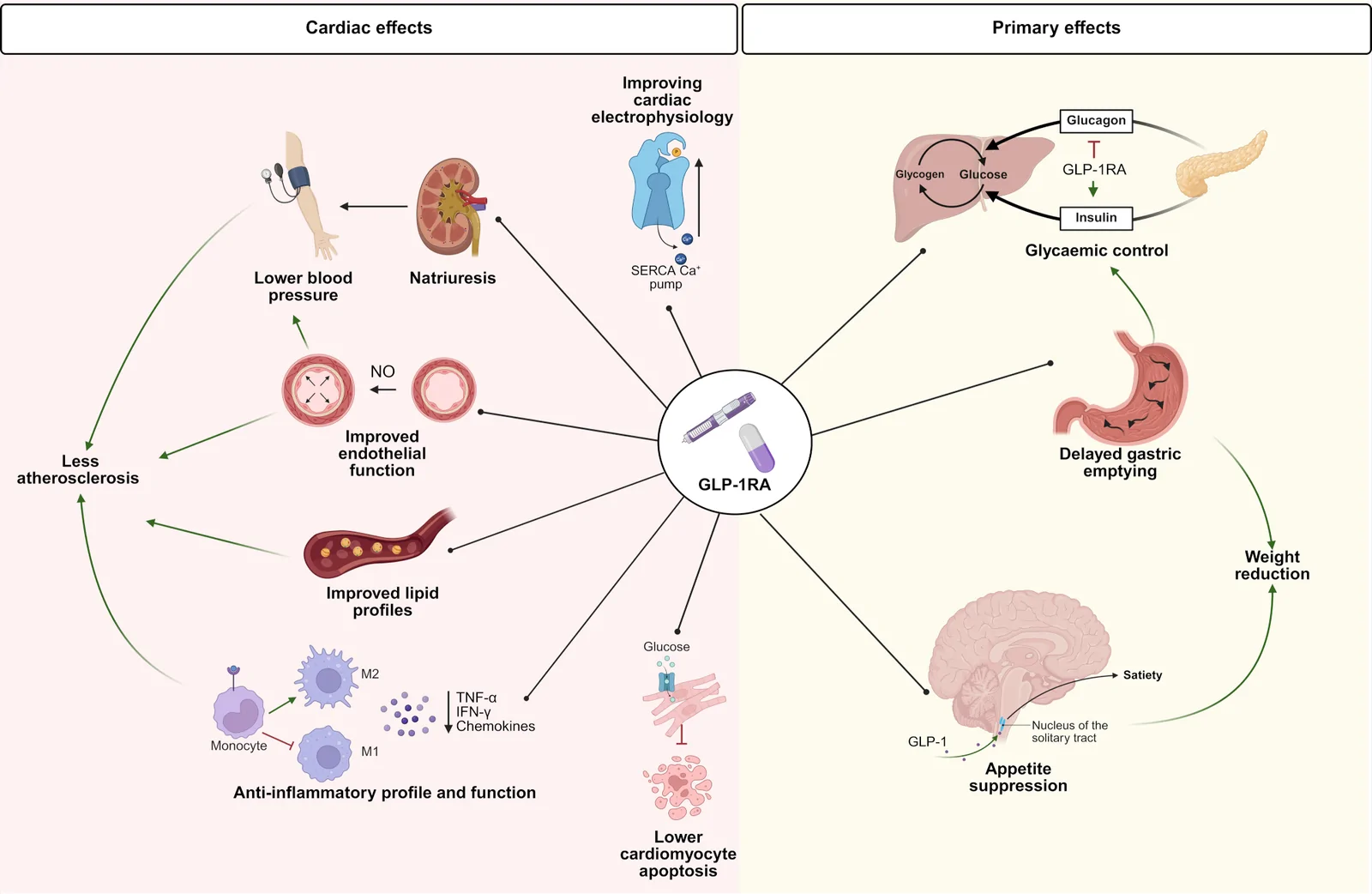
Overview of the mechanisms of action of GLP-1RAs GLP-1RAs exert primary metabolic effects by enhancing glucose-dependent insulin secretion and slowing gastric emptying, thereby improving glycaemic control. They also activate satiety pathways within the nucleus tractus solitarius and delay gastric emptying, together contributing to weight reduction. In addition to these metabolic actions, GLP-1RAs have multiple cardiac effects, including stimulation of natriuresis and reduction of blood pressure, improvement of endothelial function and lipid profiles, induction of an anti-inflammatory phenotype, regulation of cardiomyocyte apoptosis, and cardiac electrophysiology. Ca^2^⁺ *Calcium ion* GLP-1RA *Glucagon-like peptide‑1 receptor agonist *IFN‑γ *Interferon-gamma* M1 *Pro-inflammatory macrophage phenotype* M2 *Anti-inflammatory macrophage phenotype* NO *Nitric oxide* SERCA *Sarco/endoplasmic reticulum Ca*^*2*^*⁺-ATPase* TNF‑α *Tumor necrosis factor-alpha*

Overall, pharmacological GLP-1RAs mimic and amplify endogenous effects, providing therapeutic benefits in both T2D and obesity. In T2D, GLP-1RAs enhance glucose-dependent insulin secretion from pancreatic β‑cells, suppress glucagon release from α‑cells, and delay gastric emptying, thereby improving glycaemic control [8]. In both T2D and obesity, they promote weight loss through appetite suppression, delayed gastric emptying, and modulation of central satiety and food-reward pathways, and have beneficial effects on lipid metabolism, including reductions in total cholesterol levels [9]. Beyond glucose and weight regulation, GLP-1RAs exert multiple cardioprotective actions (Fig. 2; [10]).

They improve endothelial function by increasing nitric oxide (NO) production, resulting in vasodilation [11–13]. GLP-1RAs also modulate the renin-angiotensin-aldosterone system and promote natriuresis, leading to modest reductions in blood pressure [14]. Moreover, GLP-1RAs lead to improvements in lipid profiles, [15] and to lower systemic and localized inflammatory profiles [16–18]. GLP-1RAs promote M2 macrophages [16] and decrease plasma concentrations of pro-inflammatory cytokines IFN‑γ and TNF‑α and mRNA of IL‑6, CCL2, and VCAM‑1. Altogether, by inhibiting the NLRP3 inflammasome and reducing leukocyte recruitment, they potentially lead to anti-atherosclerotic effects [17, 18].

The effects of GLP-1RAs extend beyond ASCVD and may have therapeutic relevance for other cardiovascular conditions, including atrial fibrillation (AF) and heart failure with preserved ejection fraction (HFpEF). GLP-1RAs may influence cardiac electrophysiology through modulation of calcium homeostasis, [19] and may enhance myocardial glucose uptake, thereby reducing oxidative stress and cardiomyocyte apoptosis [20]. Experimental studies suggest that GLP-1RA signalling may also reduce aortic valvular calcification, although clinical evidence supporting a reduction in aortic valve stenosis risk remains limited [21, 22].

More recently, GLP-1RAs have been combined with glucose-dependent insulinotropic peptide (GIP) receptor agonists, yielding dual agonists such as tirzepatide. GIP, like GLP‑1, enhances glucose-dependent insulin secretion via pancreatic β‑cell receptors, although its insulinotropic effect is attenuated in T2D, limiting its efficacy as monotherapy. In contrast to GLP-1-mediated suppression of glucagon during hyperglycaemia, GIP may stimulate glucagon release during hypoglycaemia, contributing to protection against hypoglycaemic events. Beyond pancreatic effects, GLP‑1 reduces appetite via central pathways, whereas GIP influences central metabolic signalling and adipose tissue function. Dual GIP and GLP‑1 receptor agonism integrates these complementary mechanisms, resulting in improved glycaemic control, substantial weight loss, and favourable cardiometabolic effects [23].

## GLP-1RAs for cardiovascular prevention

Multiple large cardiovascular-outcome trials have evaluated the safety and efficacy of GLP-1RAs in individuals with T2D or obesity (Tab. 1), a population at elevated risk of CVD. These trials have consistently demonstrated cardiovascular safety with respect to major adverse cardiovascular events (3-MACE; a composite endpoint of cardiovascular death, nonfatal myocardial infarction, or nonfatal stroke, or 4‑MACE, also including hospitalization for unstable angina), with several agents showing superiority over placebo.

**Table 1:** Overview of trials where GLP‑1 receptor agonists were superior to placebo when evaluating their effects on MACE in patients with T2D or obesity

Trial	GLP-1RA	T2D	Obesity	Sample size (N)	Hazard ratio (95% CI)	P‑value (superiority)
AMPLITUDE‑O, 2021 [29]	Efpeglenatide 4 or 6 mg, once weekly subcutaneous	●		4076	0.73 (0.58–0.92)	0.007
HARMONY OUTCOMES, 2018 [27]	Albiglutide 30–50 mg, once weekly subcutaneous	●		9463	0.78 (0.68–0.90)	0.0006
LEADER, 2016 [25]	Liraglutide, 1.8 mg, once weekly subcutaneous	●		9340	0.87 (0.78–0.97)	0.01
REWIND, 2019 [28]	Dulaglutide, 0.75 or 1.5 mg, once weekly subcutaneous	●		9901	0.88 (0.79–0.99)	0.026
SELECT, 2023 [36]	Semaglutide, 2.4 mg, once weekly subcutaneous		●	17,604	0.8 (0.72–0.90)	0.001
SOUL, 2025 [30]	Semaglutide, 14 mg daily oral	●		9650	0.86 (0.77–0.96)	0.006
SUSTAIN‑6, 2016 [26]	Semaglutide, 0.5 mg or 1.0 mg once weekly subcutaneous	●		3297	0.74 (0.58–0.95)	0.02

*GLP-1RA* Glucagon-like peptide‑1 receptor agonist; *T2D* Type 2 Diabetes

### GLP-1RAs in patients with type 2 diabetes

Among the exendin-4-based GLP-1RAs, lixisenatide (10–20 µg daily) and extended-release exenatide (2 mg once weekly) were neutral for MACE (non-inferior to placebo), whereas other GLP-1RAs, liraglutide (1.8 mg daily), semaglutide (subcutaneous 0.5–1.0 mg weekly), albiglutide (30–50 mg weekly), dulaglutide (1.5 mg weekly), and efpeglenatide (4 or 6 mg weekly), significantly reduced MACE (Tab. 1, [24–30]). Oral semaglutide showed cardiovascular superiority in doses of 3–14 mg [31], supporting an oral route as a feasible option for individuals with T2D receiving complex polypharmacy.

Meta-analytic evidence indicates that GLP-1RAs reduce MACE by approximately 14%, all-cause mortality by 12%, and heart-failure hospitalizations by 14%, while also lowering body weight (−3.4 kg in PIONEER‑6 and -4.9 kg in SUSTAIN-6), HbA1c, and systolic blood pressure. These benefits are observed in both primary and secondary cardiovascular prevention populations [32].

Mediation analyses demonstrate that traditional risk factor changes explain only part of the cardiovascular benefits of GLP-1RAs, with HbA1c and urine albumin-creatinine ratio mediating at most ~65% of dulaglutide’s effect [33] ~56% of liraglutide’s, and ~64% of semaglutide’s effects [34], while albiglutide reduced MACE by 22% despite minimal weight change[28].

Growing evidence therefore supports the notion that GLP-1RAs exert vascular and anti-inflammatory effects that extend beyond weight reduction and glucose control. Across multiple trials, semaglutide has consistently demonstrated reductions in circulating inflammatory markers. A meta-analysis pooling SUSTAIN‑3 (subcutaneous semaglutide 1.0 mg vs. exenatide 2.0 mg weekly) and PIONEER‑1, -2, -5 (oral semaglutide 14 mg vs. 7 mg, empagliflozin 25 mg, or placebo daily) showed significant decreases in high-sensitivity C‑reactive protein (hsCRP), only partially mediated by changes in HbA1c and body weight [35]. These findings were supported by the SOUL trial, in which individuals with T2D and established CVD or chronic kidney disease (CKD) who received oral semaglutide had lower hsCRP concentrations than those receiving placebo (geometric mean 1.56 vs. 2.01 mg/L) [31]. Consistently, the STEP2 trial in individuals with overweight or obesity and T2D also showed modest declines in hsCRP with semaglutide 2.4 mg and 1.0 mg [36].

Although GLP-1RAs generally reduce composite MACE, heterogeneity exists in individual event subtypes. Such differences may reflect trial-level variation, including baseline risk (HbA1c levels, BMI, CVD history), demographic composition (sex, ethnicity), and event adjudication. This highlights the importance of baseline risk stratification and the development of precision-medicine strategies.

### GLP-1RAs in patients with overweight or obesity without T2D

Cardioprotective effects of GLP-1RAs have also been demonstrated in populations with overweight or obesity without T2D (Tab. 1). The largest trial on cardiovascular outcomes in patients with obesity without T2D is the SELECT trial. This randomized controlled trial showed that weekly subcutaneous semaglutide (2.4 mg) reduced the incidence of MACE by 20% compared with placebo in patients with a BMI ≥ 27 kg/m^2^ and pre-existing CVD without T2D (HR, 0.80; 95% CI, 0.72–0.90). Furthermore, subanalysis of this trial showed that improvements in cardiovascular outcomes were not solely attributable to reductions in body weight or waist circumference, again indicating that the benefits of GLP-1RAs in cardiovascular patients extend beyond weight loss alone. Other potential contributors include improvements in lipids, blood pressure, and inflammation, a factor increasingly recognised as a potential contributor to the residual CVD risk [37].

### GLP-1RAs in patients with heart failure

Emerging evidence has highlighted the relevance of GLP-1RAs in patients with heart failure (HF). Subanalysis of larger trials have predominantly focused on patients with HFpEF or mildly reduced ejection fraction (HFmrEF), as these phenotypes most commonly coexist with or are partly driven by obesity. The largest trials in patients with HFpEF include two semaglutide studies, STEP-HFpEF and STEP-HFpEF DM, as well as the SUMMIT trial with tirzepatide (Tab. 2).

**Table 2:** Overview of trials where GLP‑1 receptor agonists were superior to placebo evaluating the effect on heart failure with preserved ejection fraction

Trial	GLP-1RA	T2D	Obesity	Sample size (N)	Left ventricular function	Primary endpoint	Summary of outcomes
STEP-HFpEF, 2023 [37]	Semaglutide 2.4 mg once weekly, subcutaneous		●	529	EF > 45%	Change in KCCQ-CSS*	Estimated difference, 7.8 points; [95% CI, 4.8–10.9; *p* < 0.001]
STEP-HFpEF DM, 2024 [38]	Semaglutide 2.4 mg once weekly, subcutaneous	●	●	616	EF > 45%	Change in KCCQ-CSS*	Estimated difference, 7.3 points; [95% CI, 4.1–10.4; *p* < 0.001]
SUMMIT, 2024 [39]	Tirzepatide 2.5 mg once weekly, subcutaneous		●	731	EF > 50%	A composite of adjudicated death from cardiovascular causes or a worsening heart-failure event	HR 0.62; [95% CI, 0.41–0.95; *p* = 0.026]

^*^KCCQ-CSS Kansas City Cardiomyopathy Questionnaire clinical summary score (KCCQ-CSS; scores range from 0 to 100, with higher scores indicating better quality of life)

*EF* Ejection fraction; *GLP-1RA* Glucagon-like peptide‑1 receptor agonist; *T2D* Type 2 Diabetes

STEP-HFpEF (HFpEF without T2D) and STEP-HFpEF DM (HFpEF in T2D patients) evaluated the effect of once weekly 2.4 mg semaglutide for a year on a clinical score (KCCQ-CSS); a higher score indicates fewer symptoms and less physical limitation. A significant rise in this score was seen in patients using GLP-1RAs (estimated difference 7.3 points, 95% CI, 4.1–10.4; P < 0.001 and estimated difference 7.8 points, 95% CI, 4.8-10.9; P < 0.001), in patients with and without T2D [38, 39]. Whether these improvements translate into long-term clinical benefits requires further investigation.

The SUMMIT trial was designed to evaluate the effect of tirzepatide on major adverse HF outcomes: a composite endpoint of death from any cause and worsening HF events. Tirzepatide treatment reduced the risk on this composite endpoint (HR, 0.62; 95% CI, 0.41–0.95; *P* = 0.026) [40]. Additionally, as a secondary endpoint, a significant decrease in hsCRP level was seen at 52 weeks when using tirzepatide (between-group difference, −34.9 percentage points; 95% CI, −45.6–−22.2; P < 0.001), providing further evidence for its anti-inflammatory effects.

A meta-analysis of six large trials in patients with HFmrEF or HFpEF, including STEP-HFpEF and SUMMIT, demonstrated a significant reduction in HF events and a composite endpoint of cardiovascular death and worsening of HF (HR, 0.68; 95% CI, 0.51–0.89; *P* = 0.006, I^2^ = 47%) [41]. However, the reduction in HF hospitalizations was most pronounced in patients with obesity, suggesting that weight loss contributes importantly to the beneficial effects of GLP-1RAs in HFpEF. In particular, reductions in visceral adiposity may be relevant, as visceral fat is closely linked to systemic inflammation, a key contributor to HFpEF pathophysiology [42].

Although a minimum BMI threshold is often used as an eligibility criterion, the waist-to-height ratio, which relies on waist circumference rather than body weight, may be a more reliable indicator of visceral adiposity and its associated inflammatory burden, particularly in individuals who have a normal body weight but an elevated ratio.

The role of GLP-1RAs in heart failure with reduced ejection fraction (HFrEF) remains uncertain. The FIGHT trial, a randomized controlled trial of patients with HFrEF, showed that liraglutide did not improve LVEF or clinical stability following hospitalization for HF (19 [12%] in the liraglutide group vs 16 [11%] in the placebo group; HR, 1.10; 95% CI, 0.57–2.14; P = 0.78) and even suggested potential harm. These findings contrast with the more favourable results observed in patients with HFpEF and suggest that the benefits of GLP-1RAs may differ across HF phenotypes. However, given the relatively small sample size of the FIGHT trial, larger studies are needed to further clarify the effects of GLP-1RAs in patients with HFrEF [43].

### Other cardiometabolic effects of GLP-1RAs

Beyond patients with T2D or obesity and established CVD, GLP-1RAs have demonstrated kidney-protective effects, including a 24% lower risk of kidney disease events and a slowing of the decline in kidney function among those with and without CKD [44, 45]. Furthermore, in patients with T2D and symptomatic peripheral artery disease, weekly semaglutide 1.0 mg improved maximum walking distance by 13% relative to placebo, highlighting clinically meaningful benefits on functional capacity [46]. While studies on GLP-1RAs on AF remain scarce, GLP-1RAs also seem to be associated with a lower risk of incident and recurrent AF after ablation in patients with T2D and obesity, despite initial concerns due to the significant increase in resting heart rate observed in patients initiating therapy [47, 48]. Growing evidence supports the benefits of lifestyle-induced weight loss in atrial fibrillation management [49]. Whether pharmacologically induced weight loss with GLP-1RAs confers similar broader benefits remains uncertain. The ongoing DUTCH-WAIST trial (NCT06184633) is evaluating whether semaglutide 2.4 mg improves rhythm control compared with placebo in patients with obesity and persistent AF undergoing planned cardioversion. The therapeutic scope of GLP-1RAs continues to expand beyond cardiometabolic disease.

## The safety of GLP-1RAs

Long-term safety is an important consideration for GLP-1RAs. Since these agents are increasingly used in highly comorbid patient populations with extensive polypharmacy, and given their broad, systemic benefits, their potential for adverse effects and interdrug variability warrants attention. However, GLP-1RAs have consistently demonstrated a favourable safety profile, and in a recent meta-analysis, no differences in adverse events were observed, and serious adverse events were even reduced in patients using GLP-1RAs compared with control groups [50].

A frequently mentioned limitation of the use of GLP-1RAs is the relatively high incidence of gastrointestinal side effects, which also remains one of the most common reasons for discontinuation. An incidence of 47% to 84% of gastrointestinal-related adverse events was observed in patients using GLP-1RAs [51]. Symptoms noted were nausea, vomiting, diarrhoea, and constipation. Still, most of the symptoms were transient and of mild-to-moderate severity.

A large concern was raised when several pre-clinical studies suggested a possible association between the use of GLP-1RAs and an increased risk of thyroid C‑cell tumours [52]. However, this association has not been confirmed in clinical trials published to date. Additionally, there was caution regarding hypoglycaemia in patients without diabetes on GLP-1RAs. However, hypoglycaemia did not occur more frequently in patients without diabetes compared to those with diabetes. Other adverse outcomes mentioned in relation to GLP-1RAs include acute pancreatitis, retinopathy, arthritis, and sleep disturbances [53]. Unlike the gastrointestinal adverse effects, safety analyses have not demonstrated consistent associations for these side effects. In particular, systematic reviews and meta-analyses of randomized trials have not shown an increased risk of acute pancreatitis with GLP-1RAs, and some analyses have reported numerically lower risks compared with placebo or other therapies [54, 55].

## Currently approved GLP-1RAs

Over the years, several GLP-1RAs have been approved worldwide for the treatment of T2D and obesity. The U.S. Food and Drug Administration (FDA), as well as the Dutch Medicine Evaluation Board (‘College ter Beoordeling van Geneesmiddelen’), currently list semaglutide, liraglutide, dulaglutide, tirzepatide, lixisenatide, and exenatide as approved agents.

As discussed before, cardiovascular effects have been well established, with multiple trials already showing consistent reductions in MACE in individuals with T2D and established CVD. Consequently, European Society of Cardiology guidelines recommend GLP-1RAs for patients with chronic coronary syndrome with or without T2D [56]. European Obesity guidelines (EASO) recommend semaglutide in patients with obesity and CVD, regardless of diabetes [57]. As the GLP-1RA field advances and direct cardiovascular effects become more clearly defined, these agents are likely to play an increasingly important role in cardiovascular medicine.

The health care coverage, however, varies substantially across countries. In the Netherlands, for individuals with T2D, GLP-1RAs are reimbursed for patients with a BMI ≥ 30 kg/m^2^ who have insufficient glycaemic control despite metformin and a basal insulin, with or without a sulfonylurea derivative. Reimbursement is also available for individuals with T2D and a history of CVD and/or CKD, regardless of BMI; however, this latter indication does not apply to tirzepatide.

For individuals with obesity without T2D, reimbursement is limited to liraglutide and applies only to those with a BMI ≥ 35 kg/m^2^ with relevant comorbidities (e.g., established CVD, osteoarthritis, or sleep apnoea), or those with a BMI ≥ 40 kg/m^2^, who participated in a combined lifestyle intervention programme for at least one year [58].

## The consequences of discontinuation

Obesity is increasingly recognised as a chronic disease, and as with many other chronic conditions, pharmacotherapy may need to be continued long term. It has indeed been shown that acute discontinuation of GLP-1RA-based therapies can lead to loss of previously achieved health benefits. Most patients regain a substantial proportion of the lost weight within two years after stopping treatment, leading to renewed adiposity-driven systemic inflammation and negatively affecting cardiometabolic health. Additionally, emerging evidence indicates a rise in several cardiometabolic biomarkers following cessation, counteracting the direct positive effects conferred during treatment [59].

At the same time, emerging evidence suggests that responses after treatment cessation may not be entirely uniform across individuals. A study of tirzepatide showed a stable body weight in a smaller subgroup [60] and earlier work with liraglutide suggested that patients who participated in structured resistance training during treatment regained significantly less weight after discontinuation than those who did not receive such support [61]. Therefore, a combination of sustainable lifestyle intervention and GLP-1RAs is strongly recommended. However, these findings highlight the need for studies to identify which patients may taper or discontinue therapy, and whether dose reductions, extended dosing intervals, or lower-cost maintenance strategies can help the long-term health benefits.

## Future perspectives

GLP-1RAs offer meaningful benefits for cardiometabolic health in patients with obesity with or without T2D. However, a deeper understanding of the mechanisms by which GLP-1RAs influence cardiovascular outcomes and of the broader role of GLP-1RAs and inflammation in cardiometabolic disease is needed.

Future research should disentangle the direct cardiovascular effects of GLP-1RAs, such as their anti-inflammatory, endothelial, and natriuretic actions, from the indirect benefits mediated through weight loss and improved glucose control, particularly across phenotypes, including atherosclerosis, AF, and HF.

Several next-generation multi-receptor agonists targeting pathways such as GLP‑1, GIP, glucagon, and amylin are currently under development and are expected to produce even greater effects on body weight; their potential impact on cardiovascular outcomes will therefore be an important area of future investigation [62]. It will also be essential to determine how treatment responses differ across patient subgroups, as substantial heterogeneity is likely. The influence of metabolic profile, including HbA1c levels or BMI and waist circumference, but also ethnicity and sex differences, on outcomes with GLP-1RAs remains underexplored and warrants dedicated investigation. As the field advances, precision medicine approaches that integrate metabolic, inflammatory, genetic, and anthropometric characteristics that may help optimize cardiovascular prevention. Integrating lifestyle interventions with targeted pharmacotherapy remains essential for optimizing cardiovascular prevention.

## Data Availability

All data supporting the findings of this work are included in the article.
